# Systemic immune-inflammatory biomarkers (SII, NLR, PLR and LMR) linked to non-alcoholic fatty liver disease risk

**DOI:** 10.3389/fimmu.2024.1337241

**Published:** 2024-02-28

**Authors:** Ke Liu, Shiyun Tang, Chenhao Liu, Jianli Ma, Xiyu Cao, Xiuli Yang, Yi Zhu, Ke Chen, Ya Liu, Chuantao Zhang, Yi Liu

**Affiliations:** ^1^ Department of Respiratory Medicine, Hospital of Chengdu University of Traditional Chinese Medicine, Chengdu, Sichuan, China; ^2^ The National Clinical Trial Center of Chinese Medicine, Hospital of Chengdu University of Traditional Chinese Medicine, Chengdu, Sichuan, China; ^3^ Chengdu University of Traditional Chinese Medicine, Chengdu, Sichuan, China; ^4^ People’s Hospital of Xinjin District, Chengdu, Sichuan, China; ^5^ Department of Endocrinology, Hospital of Chengdu University of Traditional Chinese Medicine, Chengdu, Sichuan, China

**Keywords:** NAFLD, systemic immune-inflammatory biomarkers, NHANES, population-based study, metabolic disease

## Abstract

**Background:**

Systemic immune-inflammatory biomarkers including systemic immune inflammation index (SII), neutrophil-to-lymphocyte ratio (NLR), platelet-to-lymphocyte ratio (PLR), and lymphocyte-to-monocyte ratio (LMR) have been demonstrated to be associated with the risk and severity of various liver diseases. However, studies on their role and clinical significance in metabolic diseases, especially in nonalcoholic fatty liver disease (NAFLD), are limited and results are inconsistent.

**Methods:**

10821 adults aged 20 years or older were enrolled in this cross-sectional study, sourced from six cycles of the National Health and Nutrition Examination Survey (NHANES). Survey-weighted logistic regression was employed to investigate the correlation between systemic immune-inflammatory biomarkers (SII, NLR, PLR, and LMR) and NAFLD risk. Restricted cubic spline regression models and segmented regression models were used to describe nonlinear relationships and threshold effects. Subgroup and sensitivity analyses were also conducted.

**Results:**

After adjusting for all confounding variables, there was a significant positive association observed between ln-transformed SII (OR= 1.46, 95% CI: 1.27-1.69, *P <*0.001), NLR (OR= 1.25, 95% CI: 1.05-1.49, *P* =0.015), LMR (OR= 1.39, 95% CI: 1.14-1.69, *P* = 0.002) with NAFLD. A nonlinear dose-response relationship with an inverted “U”-shaped threshold of 4.64 was observed between ln(PLR) and NAFLD risk. When ln(PLR) was below 4.64, each unit increase in ln(PLR) was associated with a 0.55-fold increase in the risk of NAFLD (OR= 1.55, 95% CI: 1.05-2.31, *P <*0.05). Conversely, when ln(PLR) exceeded 4.64, each unit increase in ln(PLR) was associated with a 0.40-fold decrease in the risk of NAFLD (OR= 0.60, 95% CI. 0.44-0.81, *P <*0.05).

**Conclusion:**

ln-transformed SII, NLR, and LMR were linearly associated with NAFLD risk. ln(PLR) showed an inverted “U”-shaped nonlinear dose-response relationship with the risk of NAFLD.

## 1 Introduction

Non-alcoholic fatty liver disease (NAFLD) is one of the most common chronic liver diseases, characterized by the presence of liver fat deposition in more than 5% of hepatocytes, unrelated to excessive alcohol consumption (1, 2). It includes non-alcoholic fatty liver (NAFL) and non-alcoholic steatohepatitis (NASH), with NASH being prone to progression to liver fibrosis and potentially leading to severe complications such as liver cirrhosis, hepatocellular carcinoma, and liver failure (1, 2). NAFLD affects over 25% of the global population, with its incidence continuing to rise, making it a significant public health issue worldwide and imposing a substantial socioeconomic burden (3). It is estimated that the total population affected by NAFLD will increase by 18.3% by 2030 (4). However, awareness of the disease remains limited, as more than 95% of adult NAFLD patients are unaware of their condition (5). Cardiovascular disease (CVD) is the leading cause of mortality in NAFLD patients (6). NAFLD leads to various extrahepatic complications and is closely associated with metabolic cardiovascular risk factors such as obesity, insulin resistance, type 2 diabetes (T2DM), metabolic syndrome, hypertension, and dyslipidemia, further increasing the risk of CVD and long-term morbidity and mortality (6–8).

Although the incidence and potential risks of NAFLD are high, the pathogenesis of this disease remains incompletely understood, and there are currently no standardized and universally accepted non-invasive diagnostic methods (1, 2). NAFLD patients typically do not exhibit symptoms or may only experience fatigue and vague discomfort in the right upper abdomen, often detected through abnormal liver biochemistry or imaging examinations (9). However, studies on patients with T2DM have shown that a considerable proportion of them have normal plasma transaminase levels, even among those with clinically significant fibrosis (F2-4), with most plasma transaminase levels being below 40 U/L (10, 11). In terms of imaging examinations, ultrasound is not sufficiently sensitive for detecting mild hepatic steatosis (1, 12). However, H-MRS and MRI-PDFF are the most accurate and sensitive in diagnosing hepatic steatosis. However, their use is currently limited to clinical research due to the high cost involved (13, 14). Liver biopsy remains the gold standard for diagnosis, but its invasive nature, sampling errors, and inherent risks of complications restrict its use in clinical practice (15). Therefore, there is an urgent need to identify new and reliable biomarkers for diagnosing, prognosis, and monitoring NAFLD.

Given the complex interplay between metabolic dysfunction, chronic inflammation, and liver disease, there is increasing interest in the role of systemic inflammation in the development and progression of NAFLD (16, 17). Also, oxidative stress can mediate apoptosis and lead to inflammation by regulating Radical oxygen species (18). During the progression of NASH, there are changes in the composition of immune cells within the liver, along with interactions and disruptions between immune cells and parenchymal cells. Multiple immune cell types are involved in the development of the disease, associated with the severity of hepatic steatosis, fibrosis, inflammation, and cellular injury (19). Systemic immune-inflammatory biomarkers include the neutrophil-to-lymphocyte ratio (NLR), platelet-to-lymphocyte ratio (PLR), and lymphocyte-to-monocyte ratio (LMR), which reflect the balance of immune response and the overall inflammatory environment (20, 21). Additionally, the systemic immune inflammation index (SII) is a comprehensive novel biomarker of inflammation that reflects both localized immune responses and the overall level of inflammation in the body (22). Previous studies have reported associations between these immune-inflammatory markers and the risk and severity of various liver diseases, such as viral hepatitis, cirrhosis, and hepatocellular carcinoma (23, 24). However, there is limited research and inconsistent results regarding the role and clinical significance of SII, NLR, PLR, and LMR in NAFLD (20, 21, 25).

Therefore, The primary objective of this study was to conduct a comprehensive investigation into the association between systemic immune-inflammatory biomarkers (SII, NLR, PLR, and LMR) and the risk of NAFLD. Employing a cross-sectional study design, we utilized a substantial and representative sample from the national population to ensure a thorough analysis. The central emphasis of the study was to elucidate the potential of these markers as diagnostic indicators for NAFLD.

## 2 Methods

### 2.1 Study design and population

The National Health and Nutrition Examination Survey (NHANES), an ongoing cross-sectional study of national significance in the United States, serves as a crucial source of regular health-related data for the nation. All NHANES studies passed the National Center for Health Statistics (NCHS) Ethics Review Board and written informed consent was obtained from all participants (https://www.cdc.gov/nchs/nhanes/irba98.htm). The number of participants in the NHANES survey during the study period determined the sample size. The NHANES surveys encompass a wide array of essential domains including demographics, socioeconomic aspects, dietary patterns, and health-related information. The data collection is orchestrated using a multilevel, complex sampling methodology, further elucidated on the official NHANES website (https://www.cdc.gov/nchs/index.htm). Our study recruited 59842 participants from 6 cycles of NHANES (2017-2018, 2015-2016, 2013-2014, 2011-2012, 2009-2010, 2007-2008). To maintain the integrity and validity of our findings, stringent exclusion criteria were applied. Individuals with missing data about alcohol consumption, viral hepatitis status (serum hepatitis B surface antigen and serum hepatitis C antibody data), or essential covariates such as age, sex, ethnicity, waist circumference, fasting glucose levels, and insulin were excluded from the analysis. Pregnant and participants younger than 20 years of age were also excluded. Ultimately, the study included 10821 participants (**Supplementary Figure 1**).

### 2.2 Assessment of NAFLD

In this study, NAFLD was defined by a US Fatty Liver Index (USFLI) score exceeding 30, with careful consideration to exclude cases of excessive alcohol consumption (<20 g/day for males and <10 g/day for females) or the presence of viral hepatitis (indicated by a positive serum hepatitis B surface antigen or serum hepatitis C antibody) (26–28). The USFLI score has been validated to have an area under the operating characteristic curve (AUROC) of 0.80 (sensitivity, 62%; specificity, 88%) in diagnosing whether a subject has NAFLD (26).

### 2.3 Systemic immune-inflammatory biomarkers (SII, NLR, PLR, LMR)

Systemic immune-inflammatory biomarkers derived from complete blood count, including the SII, NLR, PLR, and LMR, have been used as predictors of risk and prognosis for various diseases (29–31). The NHANES Laboratory Procedures Manual (LPM) provides standardized protocols for measuring these biomarkers and explanations of any possible biases, details of which can be found at https://www.cdc.gov/nchs/nhanes/biospecimens/serum_plasma_urine.htm. In the present study, we sought to comprehensively unravel the correlation between systemic immune-inflammatory biomarkers and NAFLD. To achieve this, we calculated the SII, NLR, PLR, and LMR using the following formulas: SII = platelet counts × neutrophil counts/lymphocyte counts, NLR = neutrophil counts/lymphocyte counts, PLR = platelet counts/lymphocyte counts, LMR = lymphocyte counts/monocyte counts.

### 2.4 Covariates

Based on both existing literature and clinical insights, we included the following covariates: age, gender, race, family poverty income ratio (PIR), education level, smoking status, body mass index (BMI), diabetes, hypertension, hyperlipidemia, and alanine aminotransferase (ALT) (27, 28, 32). Within the NHANES survey framework, we have categorized race into five categories: Mexican American, Other Hispanic, Non-Hispanic White, Non-Hispanic Black, and Other Race. PIR was categorized as low (≤1.3), medium (1.3-3.5), and high (>3.5) based on the household poverty income ratio (27). Likewise, educational level was categorized as less than high school, high school or equivalent, and some college or more. Smoking status was determined by NHANES survey questions and participants were defined as smokers if they had smoked at least 100 cigarettes in their lifetime. BMI was categorized as <18.5, 18.5-24.9, 25.0-29.9, and ≥30.0 kg/m2. For diabetes, we adopted a comprehensive definition encompassing a fasting blood glucose level ≥126 mg/dL, a hemoglobin A1c ≥6.5%, use of oral hypoglycemic agents, insulin use, or self-reported history of diabetes (28). Hypertension was defined as a systolic blood pressure ≥140 mm Hg or diastolic blood pressure ≥90 mm Hg, or a self-reported history of hypertension or oral antihypertensive medications (32, 33). Hyperlipidemia has been defined as serum total cholesterol of 200 mg/dL, triglycerides of 150 mg/dL, high-density lipoprotein (HDL) of 40 mg/dL in men and 50 mg/dL in women, or low-density lipoprotein (LDL) of 130 mg/dL (34).

### 2.5 Statistical analyses

Continuous variables are expressed as mean (standard deviation) and categorical variables as frequency (percentage). For between-group comparisons of baseline information, weighted t-tests were used for continuous variables and weighted chi-square tests for categorical information. Since the SII, NLR, PLR, and LMR distributions were skewed, a logarithmic transformation was applied using natural logarithm (ln) to achieve an approximately normal distribution, which was then stratified into quartiles (Q1, Q2, Q3, and Q4).

First, multifactorial logistic regression was employed to analyze the influence of SII, NLR, PLR, and LMR on the risk of NAFLD. At the same time, ln-transformed SII, NLR, PLR, and LMR were considered as categorical variables (quartiles) for sensitivity analysis, and multifactorial logistic regression was repeated, with the lowest quartile (Q1) as the reference group, and the results were expressed as ratio ratios (95% confidence intervals). A trend test was also conducted. In our study, we constructed 3 models by adjusting for different confounding variables. The crude model remained unadjusted, while model 1 was adjusted for age, gender, and race. Model 2 was adjusted for all covariates based on model 1. Second, to address potential nonlinear relationships between SII, NLR, PLR, LMR, and NAFLD risk, restricted cubic spline (RCS) regression was performed. The likelihood ratio test was used to test for nonlinearity. When a nonlinear relationship was detected, a two-stage segmented regression was carried out using the inflection point values to explore the threshold effects of the independent variables on NAFLD. Further, to examine whether this relationship was modified by age, gender, race, household poverty income, education, BMI, hypertension, and hyperlipidemia, we conducted interaction analyses and subgroup analyses considering SII, NLR, PLR, and LMR as continuous and categorical variables (quartiles), respectively. Finally, as a sensitivity analysis, the fatty liver index (FLI) was utilized to validate the robustness of our results.

All data analyses were performed using R software (https://www.r-project.org/; version 4.2.1). A bilateral *P* < 0.05 was considered statistically different.

## 3 Results

### 3.1 Population characteristics

**Figure 1:**
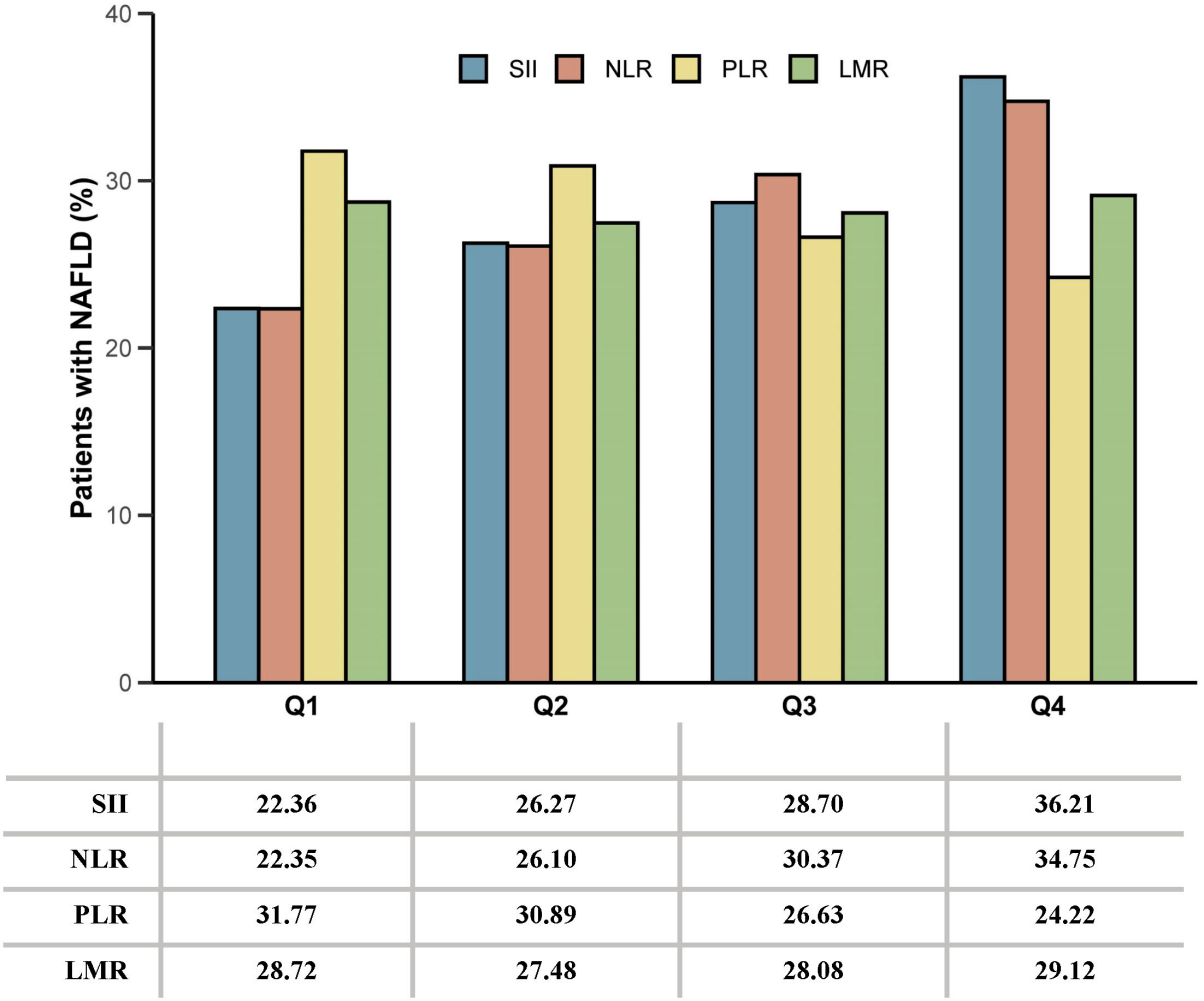
The proportion of patients with NAFLD sorted by quartiles of ln-transformed SII, NLR, PLR, and LMR. SII, systemic immune-inflammation index; NLR, neutrophil-to-lymphocyte ratio; PLR, platelet-to-lymphocyte ratio; LMR, lymphocyte-to-monocyte ratio.

A total of 10,821 subjects were enrolled in this study (**Supplementary Figure 1**). **Table 1** shows the demographic characteristics of all the participants. Among the participants, 38.48% fell within the age group of 40-59 years, and 51.00% were female. Notably, individuals with NAFLD exhibited higher household incomes, levels of education, and a higher prevalence of diabetes, hypertension, and hyperlipidemia compared to those without NAFLD. **Figure 1** shows the proportion of patients with NAFLD sorted by quartiles of ln-transformed SII, NLR, PLR, and LMR. Higher quartiles of SII and NLR were associated with a higher prevalence of NAFLD, while conversely, higher quartiles of PLR showed a lower prevalence of NAFLD. In contrast, quartiles of LMR demonstrated similar proportions of NAFLD.

**Table 1:** Characteristics of the study population.

Characteristic	Overall (n = 10821)	Non-NAFLD (n = 7496)	NAFLD (n = 3325)	*P* value
Age, n (%)				<0.001
20-39 years	3348.00 (34.81%)	2625.00 (38.67%)	723.00 (25.05%)	
40-59 years	3773.00 (38.48%)	2589.00 (38.03%)	1184.00 (39.62%)	
≥60 years	3700.00 (26.71%)	2282.00 (23.30%)	1418.00 (35.33%)	
Gender, n (%)				<0.001
Female	5522.00 (51.00%)	3964.00 (53.15%)	1558.00 (45.58%)	
Male	5299.00 (49.00%)	3532.00 (46.85%)	1767.00 (54.42%)	
Race/ethnicity, n (%)				<0.001
Mexican American	1622.00 (8.08%)	819.00 (6.14%)	803.00 (12.99%)	
Other Hispanic	1123.00 (5.36%)	737.00 (5.23%)	386.00 (5.70%)	
Non-Hispanic White	4801.00 (68.89%)	3291.00 (68.53%)	1510.00 (69.79%)	
Non-Hispanic Black	2048.00 (10.31%)	1694.00 (12.11%)	354.00 (5.76%)	
Other Race	1227.00 (7.36%)	955.00 (7.99%)	272.00 (5.76%)	
PIR, n (%)				<0.001
≤1.3	3343.00 (21.17%)	2187.00 (20.50%)	1156.00 (22.88%)	
1.3-3.5	4125.00 (36.22%)	2819.00 (34.93%)	1306.00 (39.47%)	
>3.5	3353.00 (42.61%)	2490.00 (44.57%)	863.00 (37.65%)	
Education level, n (%)				<0.001
Less than high school	2461.00 (14.99%)	1472.00 (13.21%)	989.00 (19.49%)	
High school or equivalent	2431.00 (22.45%)	1681.00 (21.88%)	750.00 (23.90%)	
Some college or more	5929.00 (62.56%)	4343.00 (64.91%)	1586.00 (56.61%)	
BMI, n (%)				<0.001
<25 kg/m^2^	2977.00 (28.67%)	2845.00 (38.68%)	132.00 (3.41%)	
25-30 kg/m^2^	3615.00 (33.12%)	2794.00 (37.55%)	821.00 (21.95%)	
≥30 kg/m^2^	4229.00 (38.21%)	1857.00 (23.77%)	2372.00 (74.65%)	
Smoking status, n (%)	4823.00 (44.35%)	3284.00 (43.74%)	1539.00 (45.88%)	0.113
Diabetes, n (%)				<0.001
No	8668.00 (85.27%)	6590.00 (91.89%)	2078.00 (68.55%)	
Yes	2153.00 (14.73%)	906.00 (8.11%)	1247.00 (31.45%)	
Hypertension, n (%)				<0.001
No	6048.00 (60.85%)	4653.00 (67.19%)	1395.00 (44.84%)	
Yes	4773.00 (39.15%)	2843.00 (32.81%)	1930.00 (55.16%)	
Hyperlipidemia, n (%)				<0.001
No	2573.00 (25.37%)	2199.00 (30.76%)	374.00 (11.77%)	
Yes	8248.00 (74.63%)	5297.00 (69.24%)	2951.00 (88.23%)	
ALT (U/L)	25.24 (17.21)	22.97 (15.25)	30.95 (20.29)	<0.001
AST (U/L)	24.96 (16.72)	24.35 (16.44)	26.51 (17.32)	<0.001
Neutrophil count (1000 cell/μL)	3.95 (1.60)	3.75 (1.55)	4.46 (1.60)	<0.001
Platelet count (1000 cell/μL)	239.92 (61.89)	237.22 (60.23)	246.73 (65.42)	<0.001
Lymphocyte count (1000 cell/μL)	2.01 (0.96)	1.96 (1.01)	2.14 (0.83)	<0.001
Monocyte count (1000 cell/μL)	0.54 (0.20)	0.52 (0.17)	0.58 (0.24)	<0.001
SII	512.30 (325.17)	493.93 (328.08)	558.65 (313.00)	<0.001
NLR	2.13 (1.09)	2.08 (1.06)	2.27 (1.14)	<0.001
PLR	129.77 (48.82)	131.84 (50.07)	124.55 (45.11)	<0.001
LMR	4.00 (1.58)	3.99 (1.55)	4.02 (1.65)	0.417

PIR, family poverty income ratio; BMI, body mass index; ALT, alanine aminotransferase; AST, aspartate aminotransferase; SII, systemic immune-inflammation index; NLR, neutrophil-to-lymphocyte ratio; PLR, platelet-to-lymphocyte ratio; LMR, lymphocyte-to-monocyte ratio.

### 3.2 Association of SII, NLR, PLR, and LMR with NAFLD risk

**Table 2** shows the relationship between SII, NLR, PLR, LMR, and risk of NAFLD. We constructed three models by adjusting for different confounding variables to evaluate the relationship between SII, NLR, PLR, LMR, and NAFLD risk. After adjusting for all confounding variables (model 2), there was a significant positive association observed between ln-transformed SII (OR= 1.46, 95% CI: 1.27-1.69, *P*<0.001), NLR (OR= 1.25, 95% CI: 1.05-1.49, *P* =0.015), LMR (OR= 1.39, 95% CI: 1.14-1.69, *P* = 0.002) with NAFLD prevalence. However, in the final model, the relationship between ln(PLR) (OR= 0.85, 95% CI: 0.70-1.03, *P* =0.092) and NAFLD risk was not significant. Consistent with this result, this trend was consistently observed when ln-transformed SII, NLR, PLR, and LMR were considered categorical variables (quartiles) in the sensitivity analysis. In the fully adjusted model (model 2), the risk of NAFLD increased progressively in the highest quartile group of SII, NLR, and LMR (Q4) compared with the lowest quartile group (Q1) (*P* for trend < 0.05). In addition, we observed that this trend also became meaningful when PLR was used as a quartile (*P* for trend < 0.05).

**Table 2:** The relationship between SII, NLR, PLR, and LMR and the risk of NAFLD.

Characteristic	Crude model ^a^		Model 1 ^b^		Model 2 ^c^	
	OR (95% CI)	*P* value	OR (95% CI)	*P* value	OR (95% CI)	*P* value
SII (ln-transformed)	1.66 (1.48, 1.85)	<0.001	1.61 (1.43, 1.82)	<0.001	1.46 (1.27, 1.69)	<0.001
SII (Quartile)						
Q1	Ref		Ref		Ref	
Q2	1.24 (1.09, 1.40)	<0.001	1.20 (1.06, 1.36)	0.006	1.15 (0.96, 1.37)	0.118
Q3	1.40 (1.21, 1.62)	<0.001	1.35 (1.16, 1.57)	<0.001	1.19 (0.98, 1.45)	0.074
Q4	1.97 (1.73, 2.25)	<0.001	1.91 (1.66, 2.20)	<0.001	1.69 (1.39, 2.05)	<0.001
*P* for trend		<0.001		<0.001		<0.001
NLR (ln-transformed)	1.63 (1.42, 1.88)	<0.001	1.39 (1.21, 1.61)	<0.001	1.25 (1.05, 1.49)	0.015
NLR (Quartile)						
Q1	Ref		Ref		Ref	
Q2	1.23 (1.06, 1.42)	0.007	1.13 (0.97, 1.32)	0.123	1.07 (0.87, 1.31)	0.539
Q3	1.51 (1.29, 1.77)	<0.001	1.38 (1.17, 1.63)	<0.001	1.17 (0.95, 1.43)	0.132
Q4	1.85 (1.59, 2.16)	<0.001	1.56 (1.32, 1.84)	<0.001	1.36 (1.08, 1.72)	0.010
*P* for trend		<0.001		<0.001		0.007
PLR (ln-transformed)	0.68 (0.58, 0.79)	<0.001	0.63 (0.54, 0.75)	<0.001	0.85 (0.70, 1.03)	0.092
PLR (Quartile)						
Q1	Ref		Ref		Ref	
Q2	0.96 (0.83, 1.10)	0.564	0.95 (0.83, 1.10)	0.526	1.14 (0.94, 1.37)	0.179
Q3	0.78 (0.66, 0.91)	0.003	0.76 (0.63, 0.90)	0.002	0.88 (0.69, 1.12)	0.294
Q4	0.69 (0.58, 0.81)	<0.001	0.65 (0.55, 0.76)	<0.001	0.85 (0.70, 1.03)	0.100
*P* for trend		<0.001		<0.001		0.033
LMR (ln-transformed)	1.00 (0.87, 1.14)	0.996	1.45 (1.24, 1.70)	<0.001	1.39 (1.14, 1.69)	0.002
LMR (Quartile)						
Q1	Ref		Ref		Ref	
Q2	0.94 (0.80, 1.11)	0.458	1.10 (0.93, 1.30)	0.247	1.10 (0.89, 1.37)	0.364
Q3	0.97 (0.83, 1.14)	0.699	1.22 (1.02, 1.45)	0.026	1.21 (0.98, 1.50)	0.074
Q4	1.02 (0.89, 1.17)	0.784	1.45 (1.24, 1.69)	<0.001	1.39 (1.16, 1.67)	<0.001
*P* for trend		0.701		<0.001		0.001

OR, odds ratio; CI, confidence interval; Q, quartile; SII, systemic immune-inflammation index; NLR, neutrophil-to-lymphocyte ratio; PLR, platelet-to-lymphocyte ratio; LMR, lymphocyte-to-monocyte ratio.

a The crude model was not adjusted for any covariates.

b Model 1 was adjusted for age, gender, and race.

c Model 2 was adjusted for all covariates based on model 1.

In parallel, we also analyzed the primary cell subpopulations for these cell ratios (**Supplementary Table 1**). After adjusting for all confounding variables (model 2), significant positive correlations were found between ln-transformed neutrophil count (OR= 2.62, 95% CI: 2.11-3.25, *P*<0.001), platelet count (OR= 2.39, 95% CI: 1.88-3.04, *P*<0.001), lymphocyte count (OR= 2.23, 95% CI: 1.72-2.88, *P*<0.001), monocyte count (OR= 1.45, 95% CI: 1.18-1.77, *P*<0.001) and NAFLD risk. The results remained unchanged when these cell subpopulations were used as categorizing variables.

### 3.3 Dose-response of systemic immune-inflammatory biomarkers (SII, NLR, PLR, and LMR) and NAFLD risk

**Figure 2:**
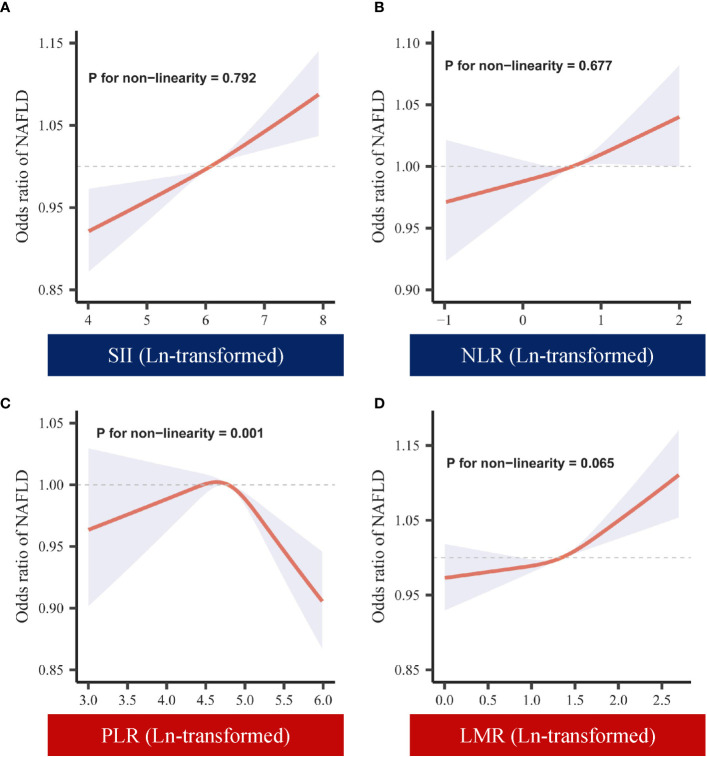
Dose-response of ln-transformed SII, NLR, PLR, LMR and NAFLD. **(A)** Dose-response of SII (ln-transformed) and NAFLD. **(B)** Dose-response of NLR (ln-transformed) and NAFLD. **(C)** Dose-response of PLR (ln-transformed) and NAFLD. **(D)** Dose-response of LMR (ln-transformed) and NAFLD. SII, systemic immune-inflammation index; NLR, neutrophil-to-lymphocyte ratio; PLR, platelet-to-lymphocyte ratio; LMR, lymphocyte-to-monocyte ratio.

To further ensure the robustness of the results, we investigated whether there was a nonlinear relationship between systemic immune-inflammatory biomarkers (SII, NLR, PLR, and LMR) and NAFLD risk. As shown in **Figure 2**, in the RCS regression model adjusting for all confounders, there was no nonlinear relationship between SII, NLR, LMR and NAFLD (*P* for nonlinearity > 0.05). This aligns with the linear regression outcomes described earlier. Interestingly, we observed an inverted “U”-shaped nonlinear dose-response relationship for PLR and the risk of NAFLD (*P* for nonlinearity < 0.05), prompting further investigation. Subsequently, in the segmented regression and threshold analysis (**Table 3**), the results showed an inflection point value of 4.64 for ln(PLR). When ln(PLR) was below 4.64, each unit increase in ln(PLR) was associated with a 0.55-fold increase in the risk of NAFLD (OR= 1.55, 95% CI: 1.05-2.31, *P <*0.05). Conversely, when ln(PLR) exceeded 4.64, each unit increase in ln(PLR) was associated with a 0.40-fold decrease in the risk of NAFLD (OR= 0.60, 95% CI. 0.44-0.81, *P <*0.05) (log-likelihood test: 0.001).

**Table 3:** The threshold effect of PLR (ln-transformed) on NAFLD was analyzed using a two-stage phased regression model.

Models	Adjusted OR (95% CI) ^a^	*P* value
Model I		
logistic regression (the standard linear model)	0.85 (0.70, 1.03)	0.092
Model II		
Inflection point	4.64	
<4.64	1.55 (1.05, 2.31)	0.029
>4.64	0.60 (0.44, 0.81)	0.001
Log likelihood ratio ^b^		0.001

OR, Odds Ratio; CI, Confidence Interval; PLR, platelet-to-lymphocyte ratio.

a Adjusted for age, gender, race, family poverty income ratio, education level, smoking status, body mass index, diabetes, hypertension, hyperlipidemia, and alanine aminotransferase.

b Model II compared to model I.

### 3.4 Subgroup analyses and sensitivity analyses

**Figure 3:**
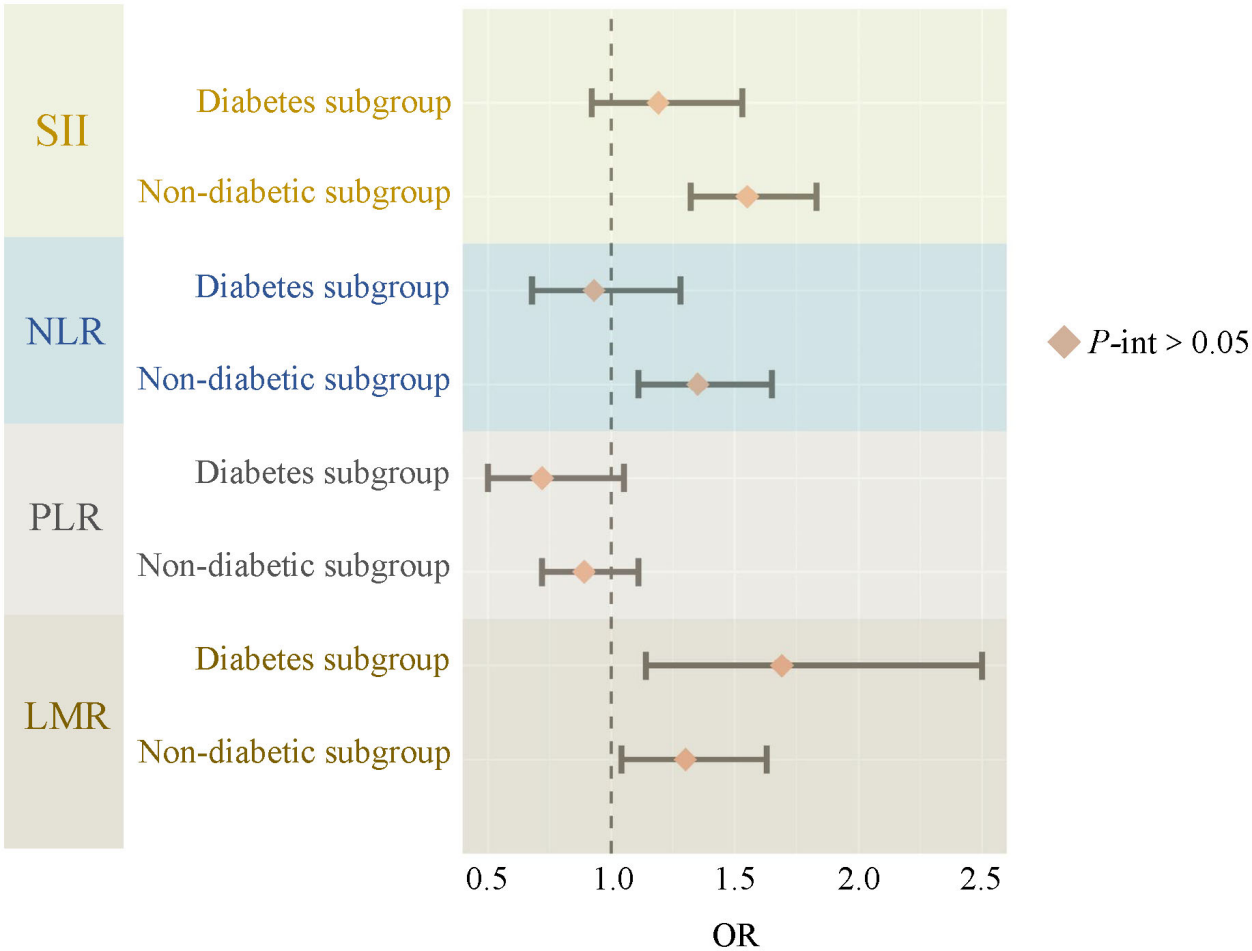
The relationship between SII, NLR, PLR, LMR and NAFLD risk in diabetes subgroups. SII, systemic immune-inflammation index; NLR, neutrophil-to-lymphocyte ratio; PLR, platelet-to-lymphocyte ratio; LMR, lymphocyte-to-monocyte ratio; P-int, P for interaction.

**Figure 3** demonstrates the relationship between systemic immune-inflammatory biomarkers (SII, NLR, PLR, and LMR) and NAFLD risk within diabetic and non-diabetic subgroups. It was found that none of the interactions between ln-transformed systemic immune-inflammatory biomarkers (SII, NLR, PLR, and LMR) and diabetes were significant (all P for interaction >0.05). Additionally, we conducted subgroup analyses for age, gender, race, PIR, education level, BMI, hypertension, and hyperlipidemia (**Table 4**). Consistently, the majority of subgroup analyses reaffirmed the lack of significant interactions (*P* for interaction >0.05). Furthermore, when ln-transformed SII, NLR, PLR, and LMR were considered as categorical variables (quartiles), we again performed subgroup analyses, and the results of all subgroup analyses similarly confirmed this finding (**Supplementary Tables 2-5**).

**Table 4:** The relationship between SII, NLR, PLR, LMR and NAFLD risk in different subgroups.

Characteristic	SII (ln-transformed)		NLR (ln-transformed)		PLR (ln-transformed)		LMR (ln-transformed)	
	OR (95% CI)	*P-int*	OR (95% CI)	*P-int*	OR (95% CI)	*P-int*	OR (95% CI)	*P-int*
Age, n (%)		0.028		0.493		0.337		0.522
20-39 years	1.76 (1.37, 2.25)		1.39 (1.02, 1.89)		1.05 (0.70, 1.58)		1.47 (1.03, 2.12)	
40-59 years	1.64 (1.25, 2.14)		1.35 (0.97, 1.89)		0.81 (0.57, 1.14)		1.40 (0.98, 1.99)	
≥60 years	1.12 (0.91, 1.38)		1.04 (0.82, 1.32)		0.75 (0.56, 1.00)		1.35 (1.02, 1.79)	
Gender, n (%)		0.464		0.341		0.058		0.192
Male	1.37 (1.15, 1.63)		1.13 (0.94, 1.37)		0.92 (0.73, 1.16)		1.33 (1.06, 1.67)	
Female	1.59 (1.29, 1.96)		1.41 (1.07, 1.87)		0.79 (0.60, 1.03)		1.42 (1.03, 1.94)	
Race/ethnicity, n (%)		0.043		0.136		0.126		0.294
Mexican American	1.15 (0.90, 1.48)		1.02 (0.77, 1.35)		0.75 (0.52, 1.09)		1.51 (1.06, 2.14)	
Other Hispanic	1.21 (0.84, 1.76)		1.23 (0.81, 1.89)		0.61 (0.35, 1.07)		1.04 (0.63, 1.74)	
Non-Hispanic White	1.43 (1.16, 1.75)		1.18 (0.91, 1.51)		0.82 (0.64, 1.05)		1.49 (1.13, 1.97)	
Non-Hispanic Black	1.46 (1.16, 1.84)		1.46 (1.11, 1.92)		0.87 (0.63, 1.21)		1.29 (0.84, 1.96)	
Other Race	2.96 (1.88, 4.66)		2.52 (1.43, 4.46)		1.84 (1.02, 3.31)		0.83 (0.46, 1.49)	
PIR, n (%)		0.910		0.906		0.553		0.042
<=1.3	1.47 (1.18, 1.83)		1.23 (0.93, 1.61)		0.99 (0.74, 1.31)		1.87 (1.38, 2.55)	
1.3-3.5	1.45 (1.20, 1.75)		1.27 (0.97, 1.65)		0.83 (0.62, 1.13)		1.27 (0.94, 1.73)	
>3.5	1.46 (1.11, 1.92)		1.22 (0.89, 1.68)		0.78 (0.54, 1.13)		1.26 (0.87, 1.83)	
Education level, n (%)		0.362		0.217		0.283		0.194
Less than high school	1.57 (1.24, 2.00)		1.47 (1.11, 1.95)		0.98 (0.67, 1.45)		1.33 (0.90, 1.96)	
High school or equivalent	1.45 (1.11, 1.91)		1.33 (0.97, 1.84)		0.91 (0.62, 1.34)		1.76 (1.19, 2.61)	
Some college or more	1.43 (1.17, 1.75)		1.16 (0.92, 1.47)		0.80 (0.62, 1.05)		1.25 (0.95, 1.63)	
Smoking status, n (%)		0.870		0.597		0.222		0.812
Yes	1.40 (1.16, 1.70)		1.22 (1.00, 1.49)		0.88 (0.68, 1.13)		1.40 (1.06, 1.83)	
No	1.50 (1.23, 1.82)		1.26 (0.98, 1.60)		0.81 (0.63, 1.05)		1.37 (1.05, 1.78)	
BMI, n (%)		0.659		0.813		0.903		0.104
<25 kg/m^2^	1.14 (0.70, 1.87)		0.98 (0.54, 1.79)		0.83 (0.43, 1.59)		1.01 (0.55, 1.86)	
25-30 kg/m^2^	1.33 (1.07, 1.65)		1.11 (0.88, 1.40)		0.90 (0.67, 1.21)		1.49 (1.07, 2.07)	
≥30 kg/m^2^	1.53 (1.26, 1.86)		1.32 (1.04, 1.69)		0.81 (0.64, 1.03)		1.38 (1.09, 1.74)	
Hypertension, n (%)		0.565		0.777		0.423		0.985
Yes	1.38 (1.13, 1.67)		1.26 (0.99, 1.60)		0.88 (0.71, 1.09)		1.36 (1.06, 1.74)	
No	1.55 (1.26, 1.91)		1.20 (0.92, 1.58)		0.79 (0.57, 1.08)		1.41 (1.05, 1.90)	
Diabetes, n (%)		0.172		0.050		0.662		0.176
Yes	1.19 (0.92, 1.53)		0.93 (0.68, 1.28)		0.72 (0.50, 1.05)		1.69 (1.14, 2.50)	
No	1.55 (1.32, 1.83)		1.35 (1.11, 1.65)		0.89 (0.72, 1.11)		1.30 (1.04, 1.63)	
Hyperlipidemia, n (%)		0.259		0.228		0.555		0.223
Yes	1.42 (1.21, 1.66)		1.20 (1.00, 1.44)		0.84 (0.69, 1.02)		1.45 (1.18, 1.78)	
No	1.65 (1.19, 2.29)		1.43 (0.91, 2.25)		0.89 (0.55, 1.46)		1.06 (0.55, 2.05)	

OR, odds ratio; CI, confidence interval; P-int, P for interaction; SII, systemic immune-inflammation index; NLR, neutrophil-to-lymphocyte ratio; PLR, platelet-to-lymphocyte ratio; LMR, lymphocyte-to-monocyte ratio; PIR, family poverty income ratio; BMI, body mass index.

In the sensitivity analysis, NAFLD was defined using an FLI score ≥60. The results (**Supplementary Table 6**) showed that in the fully adjusted model, ln-transformed SII (OR= 1.40, 95% CI: 1.24-1.58, *P <*0.001), NLR (OR= 1.18, 95% CI: 1.03-1.36, *P*=0.021), LMR (OR= 1.54, 95% CI: 1.26-1.88, *P <*0.001) remained significantly positively correlated with the prevalence of NAFLD. The association between ln(PLR) (OR= 0.92, 95% CI: 0.76-1.11, *P*=0.372) and risk of NAFLD remained non-significant (*P >*0.05). This result still supports our prior findings when ln-transformed SII, NLR, PLR, and LMR are considered categorical variables (quartiles), which indicates the robustness of our results.

## 4 Discussion

The association between systemic immune-inflammatory biomarkers and NAFLD in the American population was exhaustively examined by our research. We normalized the systemic inflammatory indices using ln-transformation. The results revealed a statistically significant positive correlation between SII, NLR, LMR, and NAFLD, underscoring that elevated values of SII, NLR, and LMR were linked to an increased risk of NAFLD. Furthermore, a nonlinear dose-response relationship was observed for PLR, characterized by an inverted “U”-shape. The nadir of NAFLD risk occurred at ln(PLR) 4.64, with a positive association observed below this threshold and a negative association above it. Further subgroup analyses showed that the associations of SII, NLR, PLR, and LMR between diabetic and nondiabetic populations with NAFLD were not significantly different, and this association was similar in other different subgroups. In conclusion, our findings suggest that SII, NLR, PLR, and LMR are strongly associated with NAFLD risk and emphasize the robustness of the findings.

The development of NAFLD is closely associated with metabolic and inflammatory disorders (35). However, the conclusions of previous relevant studies were controversial. Zhao et al. found that SII of NAFLD was non-linear associated with all-cause mortality and that an elevated SII was positively associated with reduced survival in patients with NAFLD (36). Some studies have also shown the SII was linked with NAFLD risk in a “U”-shaped pattern, and subgroup analyses showed a positive association between the SII index and the risk of NAFLD in participants without diabetes (37). Another cross-sectional study showed a nonlinear association between NLR and PLR and NAFLD, with PLR ≥ 42.29 as a protective factor of NAFLD, and NLR < 1.23 might be a risk factor of NAFLD (20). Clinical studies have shown that patients with NAFLD have higher NLR and LMR were higher than healthy controls (P<0.001), while PLR was significantly lower (38, 39). Our findings are in general agreement with the literature supporting the association of elevated systemic immune-inflammatory biomarkers with increased risk of NAFLD, demonstrating the important role of inflammation in the pathogenesis of NAFLD.

In the 2010s, 20% to 30% of the U.S. population met the criteria for NAFLD and the prevalence continues to increase, with NAFLD and NASH more prevalent in men (3, 40, 41). In contrast, our baseline results showed a similar proportion of men and women, with no significant differences seen. The largest proportion of people were aged 40-59 years in our study. A cohort study showed that risk factors, prevalence, and characteristics of NAFLD patients varied by age group (42). Consider this about the fact that senescent cells cause age-related tissue degeneration and that the accumulation of senescent cells promotes hepatic fat accumulation and steatosis. Senescence-associated mitochondrial dysfunction reduces cellular fatty acid oxidation capacity resulting in increased fat deposition capacity resulting in increased fat deposition (43). In addition, NAFLD is strongly associated with obesity, dyslipidemia, type 2 diabetes mellitus, and metabolic syndrome, which is consistent with our baseline characteristics, and patients with NAFLD have higher rates of developing diabetes mellitus, hypertension, hyperlipidemia, and higher BMI values. It may be related to the impairment of amino acid metabolism in NAFLD, and insulin resistance, which leads to the accumulation of fat in the liver, which in turn leads to a greater influx of free fatty acids into the liver, and the accumulation of fat in the liver leads to an inflammatory response (44).

Inflammatory immune response plays a key role in the development of NAFLD. In NAFLD, pro-death and other programmed deaths caused by classical or non-classical inflammasome pathways play an important role in promoting, and some cellular components released after cell death can cause a strong inflammatory response and promote the recruitment of inflammatory cells (45–47). This is consistent with our findings that elevated SII is positively associated with an increased risk of NAFLD. However, the specific mechanism needs further study. In the “two-hit hypothesis” for the progression of NAFLD, a “first hit” occurs due to liver fat accumulation and insulin resistance, resulting in a reduced sensitivity of the liver to further inflammation, leading to the development of NASH. This is associated with increased NLR and insulin resistance. Its development involves the death of hepatocytes through apoptosis and necrosis, which in turn activates macrophages, neutrophils, and pro-inflammatory pathways. The “second hit” then involves the activation of systemic pro-inflammatory pathways, particularly the increase in inflammatory cytokines, chemokines, and signaling molecules. Among them, nuclear factor-kappa B (NF-κB) and c-Jun N-terminal kinase (JNK) as the key pro-inflammatory signal molecules increased in NASH, as these signaling pathways provide a link between hepatic inflammation and insulin resistance (48). There is evidence that fat accumulation causes inflammation in the liver, activating Kupffer cells and releasing inflammatory cytokines, which enter the bloodstream and trigger a systemic inflammatory response. Qi et al. found that CXCL5 increased the lipid toxicity of hepatocytes by up-regulating NLRP3/caspase1/IL-1β signaling in KCs and exerting its pro-inflammatory properties (49). Liu et al. confirmed that CARD9 deficiency induces S100a8/a9 expression through toll-like receptors, leading to increased expression of pro-inflammatory, fibrotic, and lipid metabolism-related genes in NASH progression (50). In addition, in NAFLD, platelets are highly activated and participate in disease progression by enhancing the pro-thrombotic and pro-inflammatory states. Platelets can cause sinusoidal endothelial cells to release a large number of chemokines, increase the migration of neutrophils and lymphocytes, and induce liver injury. On the other hand, platelets can cause liver inflammation by enhancing the recruitment of white blood cells in the sinusoidal endothelium, and can further activate effector cells, thereby amplifying liver injury. Various long-term studies have shown that platelets induce the progression of NAFLD primarily by generating a pro-inflammatory and pro-fibrotic environment in the liver (51, 52). In the presence of inflammatory diseases, circulating lymphocytes are often reduced (48). The “U”-shaped nonlinear relationship between PLR and NAFLD in our study also suggests that there is a certain correlation between them. In general, the relationship between systemic immune-inflammatory biomarkers and NAFLD needs to be further studied. The significant association between systemic immune-inflammatory biomarkers and NAFLD, as well as previous literature, still indicates that systemic immune-inflammatory biomarkers participate in or may affect the occurrence of NAFLD. In any case, we know that this is still speculative and that further evidence is needed to clarify its causal relationship and mechanism of action to better make it a predictor of NAFLD risk.

Compared to earlier studies, our paper has these points. First, fewer previous studies have observed an association between systemic immune-inflammatory biomarkers and NAFLD and the results have been inconsistent (25, 36, 37, 53, 54). Our study provides new information on the quantitative relationship between the systemic immune-inflammatory biomarkers (SII, NLR, PLR, and LMR) and NAFLD indicators in the general U.S. population and explores the risk relationship between the two. Second, in contrast to previous studies, we evaluated the association between NAFLD and inflammatory markers obtained from complete blood counts (CBCs), including SII, NLR, PLR, and LMR, which is one of the most common tests used in clinical work. However, these four items have not been comprehensively addressed in previous studies and are usually analyzed as single indicators. This time, the indexes were included more comprehensively in our study, and the correlation between multiple indices and NAFLD was analyzed more specifically.

Our study also has some limitations. First, due to the cross-sectional design of NHANES, we were unable to determine a causal relationship between systemic immune-inflammatory biomarkers and NAFLD. Second, considering the potential enduring impact of inflammation on NAFLD, the utilization of anti-inflammatory drugs in certain patients prompts a need for cautious interpretation of the results. Consequently, there is an imperative for more prospective studies with larger sample sizes to further elucidate and validate these findings. In addition, the current findings may not be compelling for other racial groups because non-Hispanic whites are more predominant in NHANES. Different racial backgrounds may exhibit different prevalence rates, and these data sets may lead to regional bias. Finally, despite our meticulous consideration of multiple covariates for adjustment, the specter of residual confounding looms. The potential impact of unmeasured factors on our results cannot be entirely discounted, and there exists a risk of bias to some degree due to residual confounding.

## 5 Conclusions

In summary, findings derived from a cohort of U.S. adults indicate a noteworthy association between systemic immune-inflammatory biomarkers (SII, NLR, PLR, and LMR) and the risk of NAFLD. Specifically, elevated SII, NLR, and LMR were identified as significant contributors to an increased risk of NAFLD. Notably, the relationship between PLR and NAFLD exhibited a nonlinear pattern, suggesting a nuanced impact that is not strictly monotonic. Therefore, we emphasized the role of systemic immune-inflammatory biomarkers for NAFLD risk prediction. However, in the future, further exploration of the causal relationship between these systemic immune-inflammatory biomarkers and NAFLD is warranted.

## Data availability statement

Publicly available datasets were analyzed in this study. This data can be found here: https://www.cdc.gov/nchs/nhanes.

## Ethics statement

The studies involving humans were approved by the Centers for Disease Control and Prevention (CDC) and the National Center for Health Statistics (NCHS). The studies were conducted in accordance with the local legislation and institutional requirements. The participants provided their written informed consent to participate in this study. Written informed consent was obtained from the individual(s) for the publication of any potentially identifiable images or data included in this article.

## Author contributions

KL: Methodology, Writing – original draft, Writing – review & editing. ST: Methodology, Writing – original draft, Writing – review & editing. CL: Methodology, Writing – original draft, Writing – review & editing. JM: Data curation, Formal analysis, Writing – review & editing. XC: Data curation, Formal analysis, Writing – review & editing. XY: Data curation, Writing – review & editing. YZ: Data curation, Writing – review & editing. KC: Data curation, Writing – review & editing. YaL: Funding acquisition, Resources, Supervision, Writing – original draft, Writing – review & editing. CZ: Funding acquisition, Resources, Supervision, Writing – original draft, Writing – review & editing. YiL: Funding acquisition, Resources, Supervision, Writing – original draft, Writing – review & editing.
