# Supplementary material for: Systemic immune-inflammatory biomarkers (SII, NLR, PLR and LMR) linked to non-alcoholic fatty liver disease risk
Source: Front Immunol. 2024 Feb 28;15:1337241. doi: 10.3389/fimmu.2024.1337241 (PMC10933001; doi:10.3389/fimmu.2024.1337241)
Supplement: Supplementary file 1 [file DataSheet_1.docx]

Supplementary Material

**Supplementary Table 1** The relationship between neutrophil count, platelet count, lymphocyte count, and monocyte count and the risk of NAFLD

| **Characteristic** | **Crude model** ^a^ | |  | **Model 1** ^b^ | |  | **Model 2 ^c^** | |
| --- | --- | --- | --- | --- | --- | --- | --- | --- |
|  | **OR (95% CI)** | ***P* value** |  | **OR (95% CI)** | ***P* value** |  | **OR (95% CI)** | ***P* value** |
| Neutrophil count (ln-transformed) | 3.87 (3.33, 4.50) | <0.001 |  | 3.97 (3.36, 4.68) | <0.001 |  | 2.62 (2.11, 3.25) | <0.001 |
| Neutrophil count (Quartile) |  |  |  |  |  |  |  |  |
| Q1 | Ref |  |  | Ref |  |  | Ref |  |
| Q2 | 1.63 (1.40, 1.90) | <0.001 |  | 1.56 (1.33, 1.83) | <0.001 |  | 1.32 (1.07, 1.63) | 0.012 |
| Q3 | 2.42 (2.08, 2.82) | <0.001 |  | 2.35 (2.01, 2.75) | <0.001 |  | 1.55 (1.24, 1.93) | <0.001 |
| Q4 | 3.79 (3.28, 4.37) | <0.001 |  | 3.79 (3.25, 4.42) | <0.001 |  | 2.52 (2.04, 3.11) | <0.001 |
| *P* for trend |  | <0.001 |  |  | <0.001 |  |  | <0.001 |
| Platelet count (ln-transformed) | 1.73 (1.45, 2.07) | <0.001 |  | 2.63 (2.16, 3.21) | <0.001 |  | 2.39 (1.88, 3.04) | <0.001 |
| Platelet count (Quartile) |  |  |  |  |  |  |  |  |
| Q1 | Ref |  |  | Ref |  |  | Ref |  |
| Q2 | 0.86 (0.73, 1.03) | 0.099 |  | 0.96 (0.80, 1.14) | 0.636 |  | 0.97 (0.78, 1.22) | 0.799 |
| Q3 | 1.02 (0.88, 1.18) | 0.795 |  | 1.18 (1.02, 1.37) | 0.026 |  | 1.15 (0.95, 1.40) | 0.146 |
| Q4 | 1.44 (1.25, 1.65) | <0.001 |  | 1.89 (1.62, 2.20) | <0.001 |  | 1.75 (1.45, 2.12) | <0.001 |
| *P* for trend |  | <0.001 |  |  | <0.001 |  |  | <0.001 |
| Lymphocyte count (ln-transformed) | 2.36 (1.94, 2.87) | <0.001 |  | 3.44 (2.78, 4.27) | <0.001 |  | 2.23 (1.72, 2.88) | <0.001 |
| Lymphocyte count (Quartile) |  |  |  |  |  |  |  |  |
| Q1 | Ref |  |  | Ref |  |  | Ref |  |
| Q2 | 1.38 (1.14, 1.66) | 0.001 |  | 1.55 (1.27, 1.89) | <0.001 |  | 1.27 (1.03, 1.57) | 0.024 |
| Q3 | 1.68 (1.44, 1.96) | <0.001 |  | 2.08 (1.77, 2.43) | <0.001 |  | 1.72 (1.40, 2.12) | <0.001 |
| Q4 | 2.06 (1.75, 2.44) | <0.001 |  | 2.78 (2.33, 3.31) | <0.001 |  | 2.00 (1.60, 2.49) | <0.001 |
| *P* for trend |  | <0.001 |  |  | <0.001 |  |  | <0.001 |
| Monocyte count (ln-transformed) | 2.30 (1.96, 2.70) | <0.001 |  | 2.08 (1.74, 2.48) | <0.001 |  | 1.45 (1.18, 1.77) | <0.001 |
| Monocyte count (Quartile) |  |  |  |  |  |  |  |  |
| Q1 | Ref |  |  | Ref |  |  | Ref |  |
| Q2 | 1.31 (1.11, 1.54) | 0.001 |  | 1.25 (1.06, 1.48) | 0.008 |  | 1.04 (0.87, 1.24) | 0.675 |
| Q3 | 1.62 (1.37, 1.92) | <0.001 |  | 1.52 (1.28, 1.80) | <0.001 |  | 1.16 (0.95, 1.42) | 0.154 |
| Q4 | 1.98 (1.72, 2.27) | <0.001 |  | 1.82 (1.55, 2.12) | <0.001 |  | 1.31 (1.11, 1.56) | 0.002 |
| *P* for trend |  | <0.001 |  |  | <0.001 |  |  | 0.002 |

Abbreviations: OR, odds ratio; CI, confidence interval; Q, quartile.

^a^ The crude model was not adjusted for any covariates.

^b^ Model 1 was adjusted for age, gender, and race.

^c^ Model 2 was adjusted for all covariates based on model 1.

**Supplementary Table 2** The relationship between SII (quartile) and NAFLD risk in different subgroups

| **Characteristic** | **Q1** |  | **Q2** |  | **Q3** |  | **Q4** | ***P*-t** | ***P*-int** |
| --- | --- | --- | --- | --- | --- | --- | --- | --- | --- |
|  | **OR (95% CI)** |  | **OR (95% CI)** |  | **OR (95% CI)** |  | **OR (95% CI)** |  |  |
| Age, n (%) |  |  |  |  |  |  |  |  |  |
| 20-39 years | Ref |  | 1.25 (0.91, 1.72) |  | 1.45 (1.05, 2.00) |  | 1.94 (1.41, 2.68) | <0.001 | 0.387 |
| 40-59 years | Ref |  | 1.06 (0.78, 1.43) |  | 1.11 (0.80, 1.55) |  | 1.82 (1.28, 2.59) | 0.002 |  |
| ≥60 years | Ref |  | 1.20 (0.89, 1.61) |  | 1.06 (0.75, 1.50) |  | 1.28 (0.93, 1.76) | 0.237 |  |
| Gender, n (%) |  |  |  |  |  |  |  |  |  |
| Female | Ref |  | 1.19 (0.84, 1.69) |  | 1.25 (0.92, 1.71) |  | 1.85 (1.34, 2.55) | <0.001 | 0.944 |
| Male | Ref |  | 1.15 (0.91, 1.43) |  | 1.20 (0.91, 1.57) |  | 1.56 (1.22, 2.00) | 0.001 |  |
| Race/ethnicity, n (%) |  |  |  |  |  |  |  |  |  |
| Mexican American | Ref |  | 0.91 (0.61, 1.38) |  | 0.92 (0.62, 1.36) |  | 1.12 (0.76, 1.64) | 0.571 | 0.206 |
| Other Hispanic | Ref |  | 1.22 (0.70, 2.14) |  | 0.91 (0.58, 1.43) |  | 1.27 (0.69, 2.32) | 0.645 |  |
| Non-Hispanic White | Ref |  | 1.07 (0.85, 1.36) |  | 1.16 (0.87, 1.54) |  | 1.63 (1.23, 2.16) | 0.002 |  |
| Non-Hispanic Black | Ref |  | 2.02 (1.38, 2.94) |  | 1.44 (0.94, 2.21) |  | 1.85 (1.25, 2.74) | 0.025 |  |
| Other Race | Ref |  | 1.57 (0.72, 3.41) |  | 2.25 (1.17, 4.32) |  | 4.11 (2.10, 8.03) | <0.001 |  |
| PIR, n (%) |  |  |  |  |  |  |  |  |  |
| ≤1.3 | Ref |  | 1.34 (0.97, 1.85) |  | 1.46 (0.99, 2.15) |  | 1.67 (1.19, 2.35) | 0.006 | 0.164 |
| 1.3-3.5 | Ref |  | 1.40 (1.05, 1.87) |  | 1.33 (0.98, 1.81) |  | 1.66 (1.27, 2.18) | 0.002 |  |
| >3.5 | Ref |  | 0.90 (0.63, 1.30) |  | 0.99 (0.68, 1.44) |  | 1.74 (1.20, 2.53) | 0.005 |  |
| Education level, n (%) |  |  |  |  |  |  |  |  |  |
| Less than high school | Ref |  | 1.22 (0.80, 1.87) |  | 1.11 (0.78, 1.59) |  | 1.72 (1.20, 2.45) | 0.010 | 0.458 |
| High school or equivalent | Ref |  | 1.50 (1.00, 2.24) |  | 1.21 (0.77, 1.91) |  | 2.02 (1.28, 3.19) | 0.012 |  |
| Some college or more | Ref |  | 1.01 (0.78, 1.32) |  | 1.21 (0.92, 1.60) |  | 1.56 (1.16, 2.09) | 0.002 |  |
| BMI, n (%) |  |  |  |  |  |  |  |  |  |
| <25 kg/m2 | Ref |  | 1.09 (0.49, 2.45) |  | 1.34 (0.59, 3.03) |  | 1.20 (0.51, 2.84) | 0.578 | 0.958 |
| 25-30 kg/m2 | Ref |  | 1.14 (0.81, 1.62) |  | 1.10 (0.77, 1.58) |  | 1.45 (0.99, 2.12) | 0.071 |  |
| ≥30 kg/m2 | Ref |  | 1.14 (0.91, 1.43) |  | 1.20 (0.96, 1.50) |  | 1.80 (1.41, 2.29) | <0.001 |  |
| Smoking status, n (%) |  |  |  |  |  |  |  |  |  |
| Yes | Ref |  | 1.27 (0.97, 1.65) |  | 1.26 (0.92, 1.73) |  | 1.62 (1.20, 2.18) | 0.004 | 0.717 |
| No | Ref |  | 1.06 (0.81, 1.40) |  | 1.15 (0.89, 1.49) |  | 1.74 (1.32, 2.29) | <0.001 |  |
| Diabetes, n (%) |  |  |  |  |  |  |  |  |  |
| Yes | Ref |  | 1.16 (0.78, 1.72) |  | 1.11 (0.76, 1.62) |  | 1.18 (0.82, 1.71) | 0.426 | 0.234 |
| No | Ref |  | 1.15 (0.91, 1.44) |  | 1.23 (0.98, 1.55) |  | 1.87 (1.48, 2.37) | <0.001 |  |
| Hypertension, n (%) |  |  |  |  |  |  |  |  |  |
| Yes | Ref |  | 1.22 (0.93, 1.61) |  | 1.10 (0.82, 1.47) |  | 1.67 (1.26, 2.22) | 0.003 | 0.573 |
| No | Ref |  | 1.05 (0.79, 1.41) |  | 1.27 (0.98, 1.63) |  | 1.66 (1.23, 2.23) | <0.001 |  |
| Hyperlipidemia, n (%) |  |  |  |  |  |  |  |  |  |
| Yes | Ref |  | 1.10 (0.90, 1.34) |  | 1.11 (0.88, 1.39) |  | 1.56 (1.25, 1.94) | 0.001 | 0.248 |
| No | Ref |  | 1.52 (0.89, 2.60) |  | 1.80 (1.11, 2.92) |  | 2.51 (1.46, 4.32) | 0.001 |  |

Abbreviations: OR, odds ratio; CI, confidence interval; Q, quartile; *P*-t, *P* for trend; *P*-int, *P* for interaction; SII, systemic immune-inflammation index; NLR, neutrophil-to-lymphocyte ratio; PLR, platelet-to-lymphocyte ratio; LMR, lymphocyte-to-monocyte ratio; PIR, family poverty income ratio; BMI, body mass index.

**Supplementary Table 3** The relationship between NLR (quartile) and NAFLD risk in different subgroups

| **Characteristic** | **Q1** |  | **Q2** |  | **Q3** |  | **Q4** | ***P*-t** | ***P*-int** |
| --- | --- | --- | --- | --- | --- | --- | --- | --- | --- |
|  | **OR (95% CI)** |  | **OR (95% CI)** |  | **OR (95% CI)** |  | **OR (95% CI)** |  |  |
| Age, n (%) |  |  |  |  |  |  |  |  |  |
| 20-39 years | Ref |  | 1.14 (0.81, 1.60) |  | 1.41 (1.02, 1.95) |  | 1.56 (1.07, 2.28) | 0.012 | 0.430 |
| 40-59 years | Ref |  | 0.99 (0.68, 1.43) |  | 1.21 (0.82, 1.77) |  | 1.38 (0.91, 2.09) | 0.083 |  |
| ≥60 years | Ref |  | 1.15 (0.83, 1.60) |  | 0.87 (0.64, 1.19) |  | 1.13 (0.83, 1.52) | 0.882 |  |
| Gender, n (%) |  |  |  |  |  |  |  |  |  |
| Female | Ref |  | 1.17 (0.88, 1.55) |  | 1.36 (1.00, 1.85) |  | 1.61 (1.15, 2.25) | 0.004 | 0.659 |
| Male | Ref |  | 0.99 (0.75, 1.31) |  | 1.03 (0.77, 1.40) |  | 1.18 (0.89, 1.57) | 0.255 |  |
| Race/ethnicity, n (%) |  |  |  |  |  |  |  |  |  |
| Mexican American | Ref |  | 1.01 (0.71, 1.44) |  | 1.04 (0.72, 1.52) |  | 1.01 (0.72, 1.42) | 0.904 | 0.201 |
| Other Hispanic | Ref |  | 0.56 (0.34, 0.92) |  | 0.88 (0.53, 1.46) |  | 1.25 (0.74, 2.10) | 0.203 |  |
| Non-Hispanic White | Ref |  | 1.05 (0.78, 1.41) |  | 1.11 (0.83, 1.48) |  | 1.27 (0.90, 1.80) | 0.157 |  |
| Non-Hispanic Black | Ref |  | 1.55 (1.06, 2.24) |  | 1.48 (0.97, 2.24) |  | 1.65 (1.12, 2.44) | 0.20 |  |
| Other Race | Ref |  | 1.55 (0.85, 2.80) |  | 2.29 (1.10, 4.79) |  | 3.38 (1.63, 7.00) | 0.001 |  |
| PIR, n (%) |  |  |  |  |  |  |  |  |  |
| ≤1.3 | Ref |  | 1.41 (1.03, 1.93) |  | 1.54 (1.09, 2.16) |  | 1.40 (0.97, 2.02) | 0.072 | 0.122 |
| 1.3-3.5 | Ref |  | 1.07 (0.79, 1.47) |  | 1.30 (0.98, 1.73) |  | 1.30 (0.92, 1.84) | 0.061 |  |
| >3.5 | Ref |  | 0.89 (0.64, 1.25) |  | 0.89 (0.61, 1.31) |  | 1.38 (0.94, 2.02) | 0.139 |  |
| Education level, n (%) |  |  |  |  |  |  |  |  |  |
| Less than high school | Ref |  | 1.51 (1.06, 2.16) |  | 1.20 (0.81, 1.78) |  | 1.85 (1.25, 2.73) | 0.009 | 0.406 |
| High school or equivalent | Ref |  | 1.20 (0.79, 1.82) |  | 1.32 (0.86, 2.04) |  | 1.38 (0.90, 2.11) | 0.127 |  |
| Some college or more | Ref |  | 0.93 (0.68, 1.26) |  | 1.10 (0.83, 1.45) |  | 1.26 (0.92, 1.73) | 0.087 |  |
| BMI, n (%) |  |  |  |  |  |  |  |  |  |
| <25 kg/m2 | Ref |  | 0.56 (0.22, 1.38) |  | 0.64 (0.27, 1.53) |  | 1.01 (0.43, 2.38) | 0.896 | 0.657 |
| 25-30 kg/m2 | Ref |  | 1.06 (0.79, 1.42) |  | 1.19 (0.86, 1.65) |  | 1.14 (0.81, 1.59) | 0.347 |  |
| ≥30 kg/m2 | Ref |  | 1.13 (0.86, 1.49) |  | 1.20 (0.93, 1.55) |  | 1.50 (1.12, 2.02) | 0.009 |  |
| Smoking status, n (%) |  |  |  |  |  |  |  |  |  |
| Yes | Ref |  | 1.41 (1.06, 1.87) |  | 1.22 (0.91, 1.64) |  | 1.42 (1.05, 1.91) | 0.059 | 0.039 |
| No | Ref |  | 0.86 (0.65, 1.13) |  | 1.15 (0.86, 1.52) |  | 1.35 (1.00, 1.82) | 0.018 |  |
| Diabetes, n (%) |  |  |  |  |  |  |  |  |  |
| Yes | Ref |  | 1.23 (0.85, 1.78) |  | 0.99 (0.66, 1.48) |  | 1.02 (0.70, 1.50) | 0.800 | 0.099 |
| No | Ref |  | 1.04 (0.81, 1.34) |  | 1.22 (0.95, 1.56) |  | 1.50 (1.14, 1.97) | 0.002 |  |
| Hypertension, n (%) |  |  |  |  |  |  |  |  |  |
| Yes | Ref |  | 1.23 (0.89, 1.71) |  | 1.12 (0.82, 1.52) |  | 1.57 (1.14, 2.16) | 0.016 | 0.142 |
| No | Ref |  | 0.93 (0.70, 1.24) |  | 1.22 (0.91, 1.65) |  | 1.15 (0.85, 1.58) | 0.186 |  |
| Hyperlipidemia, n (%) |  |  |  |  |  |  |  |  |  |
| Yes | Ref |  | 1.05 (0.84, 1.31) |  | 1.09 (0.87, 1.36) |  | 1.28 (1.00, 1.64) | 0.050 | 0.208 |
| No | Ref |  | 1.21 (0.67, 2.19) |  | 1.76 (1.04, 2.99) |  | 1.89 (1.07, 3.33) | 0.012 |  |

Abbreviations: OR, odds ratio; CI, confidence interval; Q, quartile; *P*-t, *P* for trend; *P*-int, *P* for interaction; SII, systemic immune-inflammation index; NLR, neutrophil-to-lymphocyte ratio; PLR, platelet-to-lymphocyte ratio; LMR, lymphocyte-to-monocyte ratio; PIR, family poverty income ratio; BMI, body mass index.

**Supplementary Table 4** The relationship between PLR (quartile) and NAFLD risk in different subgroups

| **Characteristic** | **Q1** |  | **Q2** |  | **Q3** |  | **Q4** | ***P*-t** | ***P*-int** |
| --- | --- | --- | --- | --- | --- | --- | --- | --- | --- |
|  | **OR (95% CI)** |  | **OR (95% CI)** |  | **OR (95% CI)** |  | **OR (95% CI)** |  |  |
| Age, n (%) |  |  |  |  |  |  |  |  |  |
| 20-39 years | Ref |  | 1.08 (0.74, 1.56) |  | 0.93 (0.62, 1.39) |  | 0.95 (0.63, 1.41) | 0.633 | 0.682 |
| 40-59 years | Ref |  | 1.29 (0.90, 1.84) |  | 0.88 (0.60, 1.28) |  | 0.90 (0.65, 1.24) | 0.209 |  |
| ≥60 years | Ref |  | 0.95 (0.65, 1.37) |  | 0.79 (0.56, 1.13) |  | 0.68 (0.50, 0.94) | 0.019 |  |
| Gender, n (%) |  |  |  |  |  |  |  |  |  |
| Female | Ref |  | 1.10 (0.85, 1.43) |  | 0.79 (0.57, 1.10) |  | 0.80 (0.61, 1.05) | 0.042 | 0.440 |
| Male | Ref |  | 1.17 (0.90, 1.51) |  | 0.97 (0.73, 1.29) |  | 0.90 (0.69, 1.18) | 0.280 |  |
| Race/ethnicity, n (%) |  |  |  |  |  |  |  |  |  |
| Mexican American | Ref |  | 0.87 (0.62, 1.22) |  | 0.83 (0.54, 1.28) |  | 0.66 (0.45, 0.97) | 0.038 | 0.660 |
| Other Hispanic | Ref |  | 1.24 (0.74, 2.07) |  | 0.80 (0.48, 1.33) |  | 0.78 (0.42, 1.42) | 0.225 |  |
| Non-Hispanic White | Ref |  | 1.12 (0.86, 1.45) |  | 0.83 (0.60, 1.15) |  | 0.81 (0.62, 1.06) | 0.050 |  |
| Non-Hispanic Black | Ref |  | 1.07 (0.74, 1.55) |  | 0.92 (0.64, 1.31) |  | 0.90 (0.63, 1.28) | 0.417 |  |
| Other Race | Ref |  | 2.08 (1.03, 4.22) |  | 1.64 (0.90, 2.99) |  | 1.88 (0.91, 3.87) | 0.116 |  |
| PIR, n (%) |  |  |  |  |  |  |  |  |  |
| ≤1.3 | Ref |  | 1.25 (0.90, 1.74) |  | 1.06 (0.77, 1.46) |  | 0.85 (0.60, 1.20) | 0.218 | 0.646 |
| 1.3-3.5 | Ref |  | 0.96 (0.71, 1.31) |  | 0.77 (0.55, 1.08) |  | 0.82 (0.61, 1.11) | 0.120 |  |
| >3.5 | Ref |  | 1.24 (0.88, 1.76) |  | 0.90 (0.61, 1.33) |  | 0.86 (0.60, 1.24) | 0.199 |  |
| Education level, n (%) |  |  |  |  |  |  |  |  |  |
| Less than high school | Ref |  | 1.04 (0.72, 1.51) |  | 0.74 (0.49, 1.13) |  | 0.94 (0.65, 1.36) | 0.397 | 0.378 |
| High school or equivalent | Ref |  | 1.19 (0.80, 1.78) |  | 1.18 (0.78, 1.79) |  | 0.80 (0.51, 1.26) | 0.326 |  |
| Some college or more | Ref |  | 1.12 (0.85, 1.46) |  | 0.81 (0.60, 1.10) |  | 0.85 (0.65, 1.10) | 0.067 |  |
| BMI, n (%) |  |  |  |  |  |  |  |  |  |
| <25 kg/m2 | Ref |  | 1.40 (0.56, 3.49) |  | 0.86 (0.30, 2.48) |  | 0.85 (0.31, 2.33) | 0.549 | 0.944 |
| 25-30 kg/m2 | Ref |  | 1.25 (0.92, 1.69) |  | 1.02 (0.71, 1.47) |  | 0.87 (0.62, 1.21) | <0.001 |  |
| ≥30 kg/m2 | Ref |  | 1.04 (0.83, 1.29) |  | 0.81 (0.63, 1.05) |  | 0.82 (0.65, 1.04) | 0.48 |  |
| Smoking status, n (%) |  |  |  |  |  |  |  |  |  |
| Yes | Ref |  | 1.17 (0.87, 1.57) |  | 0.90 (0.65, 1.26) |  | 0.87 (0.65, 1.16) | 0.139 | 0.836 |
| No | Ref |  | 1.07 (0.82, 1.41) |  | 0.85 (0.62, 1.19) |  | 0.81 (0.62, 1.07) | 0.077 |  |
| Diabetes, n (%) |  |  |  |  |  |  |  |  |  |
| Yes | Ref |  | 1.05 (0.78, 1.42) |  | 0.78 (0.52, 1.17) |  | 0.73 (0.51, 1.05) | 0.049 | 0.937 |
| No | Ref |  | 1.17 (0.94, 1.45) |  | 0.92 (0.70, 1.21) |  | 0.89 (0.71, 1.11) | 0.148 |  |
| Hypertension, n (%) |  |  |  |  |  |  |  |  |  |
| Yes | Ref |  | 1.13 (0.87, 1.47) |  | 0.92 (0.68, 1.23) |  | 0.85 (0.66, 1.09) | 0.098 | 0.951 |
| No | Ref |  | 1.13 (0.83, 1.53) |  | 0.83 (0.59, 1.17) |  | 0.83 (0.61, 1.12) | 0.098 |  |
| Hyperlipidemia, n (%) |  |  |  |  |  |  |  |  |  |
| Yes | Ref |  | 1.03 (0.85, 1.25) |  | 0.83 (0.66, 1.05) |  | 0.80 (0.65, 0.98) | 0.015 | 0.146 |
| No | Ref |  | 1.89 (1.14, 3.14) |  | 1.25 (0.70, 2.23) |  | 1.18 (0.65, 2.16) | 0.934 |  |

Abbreviations: OR, odds ratio; CI, confidence interval; Q, quartile; *P*-t, *P* for trend; *P*-int, *P* for interaction; SII, systemic immune-inflammation index; NLR, neutrophil-to-lymphocyte ratio; PLR, platelet-to-lymphocyte ratio; LMR, lymphocyte-to-monocyte ratio; PIR, family poverty income ratio; BMI, body mass index.

**Supplementary Table 5** The relationship between LMR (quartile) and NAFLD risk in different subgroups

| **Characteristic** | **Q1** |  | **Q2** |  | **Q3** |  | **Q4** | ***P*-t** | ***P*-int** |
| --- | --- | --- | --- | --- | --- | --- | --- | --- | --- |
|  | **OR (95% CI)** |  | **OR (95% CI)** |  | **OR (95% CI)** |  | **OR (95% CI)** |  |  |
| Age, n (%) |  |  |  |  |  |  |  |  |  |
| 20-39 years | Ref |  | 1.06 (0.64, 1.76) |  | 1.05 (0.67, 1.66) |  | 1.26 (0.84, 1.91) | 0.289 | 0.756 |
| 40-59 years | Ref |  | 1.24 (0.89, 1.73) |  | 1.19 (0.82, 1.72) |  | 1.44 (1.03, 2.01) | 0.064 |  |
| ≥60 years | Ref |  | 0.99 (0.70, 1.41) |  | 1.49 (1.11, 2.02) |  | 1.45 (1.02, 2.07) | 0.005 |  |
| Gender, n (%) |  |  |  |  |  |  |  |  |  |
| Female | Ref |  | 0.85 (0.58, 1.25) |  | 1.07 (0.77, 1.47) |  | 1.27 (0.93, 1.74) | 0.061 | 0.174 |
| Male | Ref |  | 1.31 (1.03, 1.68) |  | 1.29 (0.98, 1.72) |  | 1.35 (1.02, 1.78) | 0.069 |  |
| Race/ethnicity, n (%) |  |  |  |  |  |  |  |  |  |
| Mexican American | Ref |  | 1.57 (1.00, 2.47) |  | 1.53 (1.04, 2.24) |  | 1.68 (1.15, 2.45) | 0.016 | 0.182 |
| Other Hispanic | Ref |  | 0.63 (0.34, 1.17) |  | 1.18 (0.72, 1.94) |  | 0.82 (0.46, 1.47) | 0.954 |  |
| Non-Hispanic White | Ref |  | 1.13 (0.85, 1.50) |  | 1.25 (0.94, 1.65) |  | 1.56 (1.20, 2.02) | 0.002 |  |
| Non-Hispanic Black | Ref |  | 0.77 (0.49, 1.20) |  | 0.88 (0.62, 1.25) |  | 1.15 (0.76, 1.73) | 0.377 |  |
| Other Race | Ref |  | 1.20 (0.58, 2.48) |  | 0.88 (0.42, 1.84) |  | 0.81 (0.45, 1.46) | 0.340 |  |
| PIR, n (%) |  |  |  |  |  |  |  |  |  |
| ≤1.3 | Ref |  | 1.50 (1.03, 2.18) |  | 1.59 (1.12, 2.27) |  | 1.79 (1.30, 2.46) | 0.001 | 0.206 |
| 1.3-3.5 | Ref |  | 0.92 (0.64, 1.32) |  | 1.22 (0.89, 1.68) |  | 1.31 (0.99, 1.74) | 0.026 |  |
| >3.5 | Ref |  | 1.12 (0.78, 1.60) |  | 1.04 (0.68, 1.59) |  | 1.30 (0.92, 1.84) | 0.242 |  |
| Education level, n (%) |  |  |  |  |  |  |  |  |  |
| Less than high school | Ref |  | 1.12 (0.67, 1.85) |  | 1.21 (0.81, 1.81) |  | 1.17 (0.79, 1.74) | 0.389 | 0.522 |
| High school or equivalent | Ref |  | 1.42 (0.96, 2.08) |  | 1.30 (0.90, 1.89) |  | 1.82 (1.28, 2.59) | 0.006 |  |
| Some college or more | Ref |  | 0.98 (0.75, 1.30) |  | 1.17 (0.89, 1.55) |  | 1.29 (0.97, 1.73) | 0.057 |  |
| BMI, n (%) |  |  |  |  |  |  |  |  |  |
| <25 kg/m2 | Ref |  | 0.44 (0.17, 1.16) |  | 0.95 (0.42, 2.14) |  | 0.90 (0.48, 1.68) | 0.692 | 0.229 |
| 25-30 kg/m2 | Ref |  | 1.41 (0.96, 2.07) |  | 1.43 (1.01, 2.01) |  | 1.76 (1.26, 2.44) | 0.002 |  |
| ≥30 kg/m2 | Ref |  | 1.05 (0.79, 1.41) |  | 1.15 (0.87, 1.52) |  | 1.29 (1.01, 1.66) | 0.029 |  |
| Smoking status, n (%) |  |  |  |  |  |  |  |  |  |
| Yes | Ref |  | 1.17 (0.85, 1.59) |  | 1.31 (1.01, 1.71) |  | 1.42 (1.08, 1.88) | 0.016 | 0.866 |
| No | Ref |  | 1.05 (0.80, 1.37) |  | 1.10 (0.83, 1.46) |  | 1.35 (1.03, 1.76) | 0.036 |  |
| Diabetes, n (%) |  |  |  |  |  |  |  |  |  |
| Yes | Ref |  | 1.47 (0.99, 2.18) |  | 1.79 (1.20, 2.67) |  | 1.86 (1.27, 2.73) | 0.002 | 0.087 |
| No | Ref |  | 1.02 (0.81, 1.29) |  | 1.10 (0.87, 1.39) |  | 1.27 (1.05, 1.55) | 0.025 |  |
| Hypertension, n (%) |  |  |  |  |  |  |  |  |  |
| Yes | Ref |  | 1.01 (0.75, 1.35) |  | 1.33 (1.02, 1.73) |  | 1.48 (1.16, 1.89) | 0.001 | 0.301 |
| No | Ref |  | 1.19 (0.85, 1.67) |  | 1.10 (0.80, 1.52) |  | 1.33 (0.98, 1.80) | 0.117 |  |
| Hyperlipidemia, n (%) |  |  |  |  |  |  |  |  |  |
| Yes | Ref |  | 1.05 (0.84, 1.30) |  | 1.23 (0.99, 1.53) |  | 1.39 (1.15, 1.69) | 0.001 | 0.371 |
| No | Ref |  | 1.47 (0.90, 2.39) |  | 1.17 (0.63, 2.16) |  | 1.33 (0.71, 2.48) | 0.543 |  |

Abbreviations: OR, odds ratio; CI, confidence interval; Q, quartile; *P*-t, *P* for trend; *P*-int, *P* for interaction; SII, systemic immune-inflammation index; NLR, neutrophil-to-lymphocyte ratio; PLR, platelet-to-lymphocyte ratio; LMR, lymphocyte-to-monocyte ratio; PIR, family poverty income ratio; BMI, body mass index.

**Supplementary Table 6** The relationship between SII, NLR, PLR, and LMR and the risk of NAFLD (NAFLD was defined using an FLI score ≥60)

| **Characteristic** | **Crude model ^a^** | |  | **Model 1 ^b^** | |  | **Model 2 ^c^** | |
| --- | --- | --- | --- | --- | --- | --- | --- | --- |
|  | **OR** **(95% CI**) | ***P* value** |  | **OR** **(95% CI**) | ***P* value** |  | **OR** **(95% CI**) | ***P* value** |
| SII (ln-transformed) | 1.60 (1.45, 1.76) | <0.001 |  | 1.62 (1.47, 1.78) | <0.001 |  | 1.40 (1.24, 1.58) | <0.001 |
| SII (Quartile) |  |  |  |  |  |  |  |  |
| Q1 | Ref |  |  | Ref |  |  | Ref |  |
| Q2 | 1.22 (1.07, 1.40) | 0.004 |  | 1.23 (1.07, 1.41) | 0.003 |  | 1.20 (0.99, 1.46) | 0.066 |
| Q3 | 1.44 (1.26, 1.65) | <0.001 |  | 1.46 (1.27, 1.68) | <0.001 |  | 1.24 (1.02, 1.50) | 0.031 |
| Q4 | 1.88 (1.66, 2.14) | <0.001 |  | 1.92 (1.69, 2.17) | <0.001 |  | 1.59 (1.31, 1.92) | <0.001 |
| *P* for trend |  | <0.001 |  |  | <0.001 |  |  | <0.001 |
| NLR (ln-transformed) | 1.44 (1.29, 1.62) | <0.001 |  | 1.37 (1.22, 1.54) | <0.001 |  | 1.18 (1.03, 1.36) | 0.021 |
| NLR (Quartile) |  |  |  |  |  |  |  |  |
| Q1 | Ref |  |  | Ref |  |  | Ref |  |
| Q2 | 1.23 (1.06, 1.41) | 0.006 |  | 1.20 (1.04, 1.39) | 0.015 |  | 1.18 (0.94, 1.47) | 0.145 |
| Q3 | 1.46 (1.25, 1.70) | <0.001 |  | 1.43 (1.22, 1.67) | <0.001 |  | 1.18 (0.96, 1.44) | 0.109 |
| Q4 | 1.61 (1.40, 1.86) | <0.001 |  | 1.52 (1.32, 1.76) | <0.001 |  | 1.27 (1.03, 1.56) | 0.026 |
| *P* for trend |  | <0.001 |  |  | <0.001 |  |  | 0.019 |
| PLR (ln-transformed) | 0.73 (0.63, 0.84) | <0.001 |  | 0.71 (0.61, 0.82) | <0.001 |  | 0.92 (0.76, 1.11) | 0.372 |
| PLR (Quartile) |  |  |  |  |  |  |  |  |
| Q1 | Ref |  |  | Ref |  |  | Ref |  |
| Q2 | 0.98 (0.84, 1.14) | 0.800 |  | 0.99 (0.85, 1.16) | 0.901 |  | 1.16 (0.92, 1.47) | 0.210 |
| Q3 | 0.85 (0.73, 0.98) | 0.030 |  | 0.84 (0.72, 0.98) | 0.027 |  | 0.97 (0.76, 1.23) | 0.771 |
| Q4 | 0.74 (0.64, 0.86) | <0.001 |  | 0.73 (0.63, 0.84) | <0.001 |  | 0.91 (0.74, 1.12) | 0.359 |
| *P* for trend |  | <0.001 |  |  | <0.001 |  |  | 0.148 |
| LMR (ln-transformed) | 1.17 (1.02, 1.34) | 0.027 |  | 1.46 (1.26, 1.69) | <0.001 |  | 1.54 (1.26, 1.88) | <0.001 |
| LMR (Quartile) |  |  |  |  |  |  |  |  |
| Q1 | Ref |  |  | Ref |  |  | Ref |  |
| Q2 | 1.04 (0.90, 1.20) | 0.582 |  | 1.15 (0.99, 1.32) | 0.061 |  | 1.18 (0.95, 1.46) | 0.131 |
| Q3 | 1.16 (1.02, 1.32) | 0.029 |  | 1.33 (1.16, 1.52) | <0.001 |  | 1.47 (1.22, 1.78) | <0.001 |
| Q4 | 1.15 (0.99, 1.34) | 0.066 |  | 1.42 (1.21, 1.66) | <0.001 |  | 1.47 (1.19, 1.81) | <0.001 |
| *P* for trend |  | 0.025 |  |  | <0.001 |  |  | <0.001 |

Abbreviations: OR, odds ratio; CI, confidence interval; Q, quartile; SII, systemic immune-inflammation index; NLR, neutrophil-to-lymphocyte ratio; PLR, platelet-to-lymphocyte ratio; LMR, lymphocyte-to-monocyte ratio.

^a^ The crude model was not adjusted for any covariates.

^b^ Model 1 was adjusted for age, gender, and race.

^c^ Model 2 was adjusted for all covariates based on model 1.





**Supplementary Figure 1** Flow diagram of the screening of study participants. NHANES, National Health and Nutrition Examination Survey; SII, systemic immune-inflammation index; NLR, neutrophil-to-lymphocyte ratio; PLR, platelet-to-lymphocyte ratio; LMR, lymphocyte-to-monocyte ratio.
